# A New Principle for Information Storage in an Enzymatic Pathway Model

**DOI:** 10.1371/journal.pcbi.0030124

**Published:** 2007-06-22

**Authors:** Bruno Delord, Hugues Berry, Emmanuel Guigon, Stéphane Genet

**Affiliations:** 1 Institut National de la Santé et de la Recherche Médicale (INSERM), Unité 742, Université Pierre et Marie Curie-Paris 6 (UPMC), Paris, France; 2 Institut National de Recherche en Informatique et en Automatique (INRIA), Unité Futurs, Team Alchemy, Orsay, France; University College London, United Kingdom

## Abstract

Strong experimental evidence indicates that protein kinase and phosphatase (KP) cycles are critical to both the induction and maintenance of activity-dependent modifications in neurons. However, their contribution to information storage remains controversial, despite impressive modeling efforts. For instance, plasticity models based on KP cycles do not account for the maintenance of plastic modifications. Moreover, bistable KP cycle models that display memory fail to capture essential features of information storage: rapid onset, bidirectional control, graded amplitude, and finite lifetimes. Here, we show in a biophysical model that upstream activation of KP cycles, a ubiquitous mechanism, is sufficient to provide information storage with realistic induction and maintenance properties: plastic modifications are rapid, bidirectional, and graded, with finite lifetimes that are compatible with animal and human memory. The maintenance of plastic modifications relies on negligible reaction rates in basal conditions and thus depends on enzyme nonlinearity and activation properties of the activity-dependent KP cycle. Moreover, we show that information coding and memory maintenance are robust to stochastic fluctuations inherent to the molecular nature of activity-dependent KP cycle operation. This model provides a new principle for information storage where plasticity and memory emerge from a single dynamic process whose rate is controlled by neuronal activity. This principle strongly departs from the long-standing view that memory reflects stable steady states in biological systems, and offers a new perspective on memory in animals and humans.

## Author Summary

It is now widely recognized that learning and memory rely on activity-dependent plastic modifications of the synaptic and intrinsic properties of individual neurons. Experimental studies have identified numerous molecules that are necessary for the induction and the maintenance of plastic modifications, including activity-dependent kinase and phosphatase (aKP) cycles. In contrast, the mechanisms that govern information storage in neurons remain obscure. Prevailing theoretical models either account for the rapid onset (models of plasticity) or for the protracted maintenance (models of memory) of plastic modifications, but have failed to embody both properties. We show in a biophysical model that the ubiquitous upstream activation of aKP cycles by neuronal activity is sufficient to generate information storage that combines rapid induction and maintenance with lifetimes compatible with animal and human memory. Moreover, aKP cycles exhibit essential information storage properties consistent with experimental data, including bidirectional plasticity, graded memory, and robustness to stochastic molecular fluctuations. The aKP model offers a realistic unified framework in which cellular plasticity and memory can be interpreted as two modes of a single process where dynamics depends on neuronal activity. This new principle is dynamic in essence and challenges the widespread idea that memory reflects stability in biological systems.

## Introduction

Neurons continuously modify their synaptic and intrinsic membrane properties in response to variations in their environment (e.g., ionic conditions, neuromodulatory influences, and synaptic input). These adaptive modifications are set up rapidly, can be memorized from seconds to years, and subserve essential neuronal functions such as homeostatic regulation and information storage. Of particular importance, activity-dependent modifications are generally considered to provide the cellular basis for behavioral learning and memory in animals and humans [1]. At the molecular level, activity-dependent modifications implicate complex networks of densely interconnected signaling pathways and modulation of gene expression [2–4]. A central role has been attributed to protein kinase and phosphatase (KP) cycles in these networks because strong experimental evidence indicates that they exert a critical control over the induction [5–7] and maintenance [8,9] of activity-dependent plastic modifications. However, the question stands whether KP cycles constitute the genuine organization that is mechanistically responsible for memory formation, or whether information storage requires the consideration of larger interaction networks or gene regulation [2–4]. Models have largely been used to address this question because they allow us to simulate pathways of arbitrary complexity [2,10,11] and to identify which architectures and dynamics are required for realistic information storage.

Several mathematical models have addressed the potential contribution of KP cycles to the induction of plastic modifications in neurons, including models of long-term potentiation (LTP) and long-term depression (LTD). In these models, KP cycles control the strength of the excitatory synapse via the number of phosphorylated amino-3-hydroxy-5-methyl-4-isoxazolepropionic acid (AMPA) receptors [11–15]. In particular, these models predict that the direction (increase or decrease) of synaptic modifications depends on the postsynaptic calcium concentration, in agreement with experimental observations of activity or spike-timing dependences [16–19]. However, whatever their level of detail, models that account for the induction of synaptic modifications fail to provide explanatory mechanisms for the maintenance of these modifications [11–15].

Another class of KP cycle models has been investigated in which bistability (i.e., two stable levels of phosphorylated substrate) accounts for binary storage of information. Here, bistability arises from autoactivation mechanisms (i.e., positive feedback [20]), such as autophosphorylation [10,21–23] or phosphorylation loops within signaling networks [2,24]. In particular, autoactivation of the Ca2^+^/calmodulin-dependent protein kinase II (CaMKII) may underlie all-or-none LTP at individual CA3–CA1 hippocampal synapses (i.e., binary storage of synaptic information [25]). Albeit in cellular electrophysiological recordings synaptic plasticity appears as a graded phenomenon [16–18], it may be interpreted as the summation of binary plastic modifications at individual synapses [26]. However, experimental evidence indicates that plasticity can express in a graded manner at the single synapse level, such as in CA3–CA3 synapses [27]. Moreover, LTP and LTD generally involve graded regulation of the number and functional state of membrane receptors at individual synapses [28–30] and may implicate KP cycles devoid of autoactivation [31,32]. Similarly, both the so-called synaptic scaling of individual synapses [33–37] and the plasticity of intrinsic membrane properties [38–41] exhibit graded modifications. Thus, experimental evidence strongly supports the existence of local graded forms of information storage in neurons that cannot be explained by bistable models. Moreover, prevailing biophysical bistable models of synaptic memory involving CaMKII present slow rising kinetics to the stable high-phosphorylated (“on”) steady state (minutes to days) [21,22,42] that is inconsistent with the rapid induction of plasticity observed experimentally (e.g., [6,25,30,39,43]). Furthermore, bistability in these models lies in a region of calcium concentration that overlaps the basal calcium concentration. As a consequence, LTD can only be induced by lowering calcium below its basal level, so that these models do not account for LTD induction at intermediate calcium levels, a well-established experimental observation [16,17]. The implication of CaMKII itself in the maintenance of synaptic information is in fact strongly challenged [44–46], and some forms of plasticity do not implicate CaMKII, such as in parallel fiber synapses in the cerebellar cortex [47]. Most probably, information storage in neurons does not, therefore, rely exclusively on the bistability of autoactivating KP cycles.

Indeed, most KP cycles involved in learning and memory are devoid of autoactivation, but share the common feature of being activated by upstream signals that reflect neuronal activity. Such activity-dependent KP (aKP) cycles are ubiquitous and include most major kinases (e.g., protein kinase A [PKA], protein kinase C [PKC], and mitogen-activated protein kinase [MAPK]) and phosphatases (e.g., protein phosphatases PP1, PP2A, and PP2B). In the present model, we evaluate the performance of generic aKP cycles in information storage. Our results show that aKP cycles account for both induction (plasticity) and maintenance (memory) of synaptic or intrinsic modifications in neurons, through a single activity-dependent process controlled by activity. Consistent with experimental observations, plasticity induction is rapid, bidirectional, and graded in our model, as opposed to bistable models of information storage [10,21]. Mechanistically, our model predicts that plastic modifications reflect the instantaneous rate of KP cycles operation rather than their steady state, in opposition to previous models of plasticity induction [11–15,48,49]. The storage of information is reliable—even in spines where stochastic molecular fluctuations are maximal—and robust to the passage of time, with memory ranging from short to long term, depending on the biophysical properties of the model. In the presence of molecular turnover, the duration of aKP memory is limited by the time constant of turnover. This limit in turn depends on the substrate, but can increase up to weeks [50]. As an extension to our model, we have implemented a speculative mechanism originally proposed by Crick [51] to quantitatively evaluate its protective action on memory. We show that this mechanism efficiently restores memory timescales that are compatible with animal and human memory even in the presence of molecular turnover. In all cases (whether Crick's mechanism is assumed or not), memory arises as a natural property of aKP cycles because plastic modifications are maintained long-term after the offset of the stimuli that induced plasticity.

To our knowledge, the aKP model we propose is the first one to provide a coherent framework that links the induction and the maintenance of activity-dependent modifications. The present model therefore departs from previous models of plasticity induction that do not support maintenance of plastic modifications (i.e., that are devoid of memory [11–15,48,49]). It also differs deeply from infinite memory models that do not account for critical properties of plasticity induction [10,21]. Hence, the aKP cycle model we have studied fills the gap between these previous theories, and we discuss it as a new principle for the emergence of plasticity and memory.

## Results

**Figure 1:**
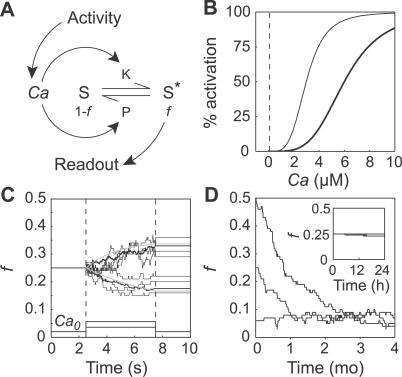
The aKP Cycle Model (A) In its macroscopic determinist approximation, the model consists of a simple reaction scheme. The nonphosphorylated (S) and phosphorylated (S*) forms are present with fractions 1 − *f* and *f*, respectively, and are interconverted with rates *K* and *P* by the kinase and a phosphatase. Enzymes are activity dependent through calcium, and *f* is the readout variable. (B) Kinase (thick line) and phosphatase (thin line) calcium activation functions. Dashed line indicates *Ca*_0_, the basal *Ca* (0.1 μM). (C) Rapid plastic modifications in *f* in response to *Ca* pulses from *Ca*_0_ to 6 μM (upper traces) and 3 μM (lower traces) in the stochastic (individual traces: thin black trace; mean trace [n = 20]: thick black trace) and deterministic (thick gray trace) models. (D) Relaxation of *f* at *Ca*_0_ from different *f* values. Inset: enlargement of three relaxation traces during 24 h illustrates the low evolution rate of *f*.

To address the contribution of aKP cycles to neuronal plasticity and memory, we devised a model (Figure 1A) where enzymes are cooperatively activated by a common molecular signal of neuronal activity (e.g., intracellular calcium; Figure 1B). Both enzymes determine the phosphorylated fraction *f* of a substrate *S*, whose dynamics are considered to embody the induction and maintenance of activity-dependent plastic modifications. *f* can be viewed as the fraction of synaptic membrane receptors or ionic channels in synaptic or intrinsic plasticity, respectively. Alternatively, the influence of *f* may be indirect, for instance through the regulation of the number of receptors or ionic channels, by determining trafficking rates in and out of the synaptic or somatic membrane. However, our goal was not to specifically identify one of the many plastic processes at work in neurons, but rather to unravel the principles governing information storage in relation with aKP operation. Individual molecular events were simulated to account for random fluctuations due to small numbers of molecules encountered in subcellular compartments. A deterministic (mean-field) approximation of the stochastic model was also studied (see Methods).

### Plasticity and Memory

In neurons, plastic modifications are rapid events compared with the timescale of their maintenance. Plastic modifications can be triggered by micromolar calcium elevations, and within seconds [6,25,30,39,43]. Simulations of our model show that aKP cycles can account for both the rapid induction and the protracted maintenance of activity-dependent modifications. Activation of the enzymes by large calcium pulses (6 μM) induces rapid increases in *f* through step-like variations (Figure 1C, upper traces). Variability in *f* values at the end of pulses reflects the stochasticity of enzyme reactions due to random molecular encounters. When the number of runs is increased, the average of stochastic simulations (Figure 1C, thick black trace) converges to the trace of the deterministic model (Figure 1C, thick gray trace), unraveling the relaxation of *f* toward a unique steady-state value, *f*_∞_, during the pulse. As observed experimentally [17], the direction (increase or decrease) of plastic modifications in the model is tightly related to the free cytosolic calcium concentration, *Ca*: *f* systematically decreases during smaller calcium pulses (3 μM; Figure 1C, lower traces). In all cases, when *Ca* is switched back to its basal level, *Ca*_0_, both enzymes inactivate almost completely (see Figure 1B), and *f* appears constant on the second timescale (Figure 1C). Longer simulations evidence that *f* needs several months to relax to its steady-state value at *Ca*_0_, *f*_∞_(*Ca*_0_) (Figure 1D). Thus, the model predicts that activity-dependent modifications can be induced rapidly in either direction and memorized long-term in aKP cycles.

### Information Storage

**Figure 2:**
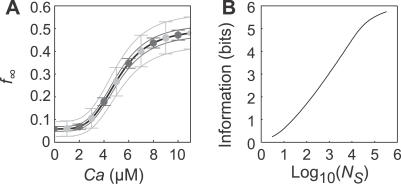
Information Coding (A) The steady-state phosphorylated fraction, *f*_∞_, is a graded function of *Ca* in the deterministic (thick black line) and stochastic models (circles; number of substrate molecules, *N_S_*, is 500 [dark gray] and 50 [light gray]). Standard deviations were derived from simulations (error bars) or theoretical probability distributions (gray lines; see Methods, Equations 16–17). (B) The information about *Ca* encoded in *f* versus *N_S_*.

The core function of plasticity and memory is information storage. The amount of information that can be stored is thus critical, and has major computational implications. In neurons, plastic modifications can be all-or-none [25] or graded [27–39,41], and we addressed the question whether aKP cycles can account for these different forms of information coding. Both stochastic simulations and the deterministic model show that the steady-state phosphorylated fraction, *f*_∞_, is an increasing sigmoid function of the calcium concentration (Figure 2A). Therefore, arbitrary graded values of the phosphorylated fraction *f* can in principle be encoded by the appropriate calcium concentrations. However, molecular reactions are stochastic in nature, and random fluctuations in small systems may blur the encoding of calcium concentrations into *f* values. To quantify this effect, we have computed *f* variability as a function of *Ca*. To this aim, we first evaluated the number of substrate molecules that may be encountered in neuronal subcellular compartments of different sizes (see Methods). We found that the standard deviation of the phosphorylated fraction, *σ_f_*, remains minute compared with its steady-state value *f*_∞_ at micromolar calcium concentrations (>2 μM), and decreases with increasing numbers of substrate molecules (see error bars in Figure 2A). Accordingly, the coefficient of variation is low, and information coding is therefore reliable in large compartments such as somata and large dendritic segments, containing more than hundreds of substrate molecules (*cv* < 0.08 for 500 substrate molecules; see Methods). Coding remains reliable even in the limit case of an individual synaptic spine (*cv* < 0.23 for 50 substrate molecules). It is noteworthy that reliability is independent of the number of enzyme molecules (see Methods, Equation 18). To quantify information coding capacity, we computed Shannon's mutual information in aKP cycles (see Methods, Equation 19). Mutual information (expressed in bits) represents the amount of information that *f* can encode about the calcium input, given a particular *Ca* distribution. Hence, it can be thought of as the number of calcium levels that can be discriminated into distinct *f* states. This measure primarily depends on the number of substrate molecules in the aKP model. In an individual spine, one to three bits of information can be encoded (i.e., up to eight distinct calcium levels can be discriminated; Figure 2B). This encompasses binary as well as coarse-grained graded coding. In somata and large dendritic segments, storage capacity is larger (three to six bits), and graded coding approximates a continuous function, with a few dozen calcium levels being discriminated (Figure 2B). This result is consistent with the experimental observation that plastic modifications of somatic and dendritic currents allow the storage of dozens of distinct states in entorhinal pyramidal neurons [52]. Therefore, in the model, aKP cycles can achieve binary to coarse-grained coding in individual spines and graded coding in somata or dendritic compartments.

### Temporal Properties

**Figure 3:**
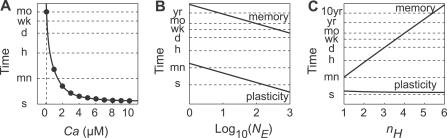
Temporal Properties (A) The time constant of *f*, *τ_f_*, as a function of *Ca* in the deterministic (line) and stochastic models (circles, *n* = 20). Dashed line: *Ca*_0_. (B) Time constants for plasticity and memory (see Methods) versus the number of enzyme molecules, *N_E_*. (C) Time constants versus the Hill number for *Ca* activation *n_H._*. In (B) and (C), all other parameters are kept at their standard value in the deterministic model (see Methods).

In neurons, the characteristic time for plasticity induction ranges from a fraction of a second to dozens of seconds ([6,19,25,28,30,32,39–41,43,53–56]; see Discussion). In contrast, storage lasts from seconds to months or more, depending on the neuronal type and function considered ([8,29,54,55,57–62]; see Discussion). Our model shows that aKP cycles account for these temporal properties. Dynamics of the aKP cycle (i.e., the evolution rate of the phosphorylated fraction *f*) is directly governed by calcium concentration (Figure 3A). *f* dynamics is set by a single time constant, τ*_f_*, which covers a wide range that spans six orders of magnitude (Figure 3A). Using micromolar calcium signals that are known to induce plasticity experimentally, *τ_f_* is on the order of a second with standard parameter values (Figure 3A), and thus plastic modifications are rapid (see Figure 1C), consistent with experimental observation. Below 1 μM, *τ_f_* increases stiffly and aKP dynamics are much slower. This results in memory lifetimes that culminate at ~1 mo at resting calcium *Ca*_0_ (see also Figure 1D). We assessed the influence of molecular characteristics on these temporal properties. The kinetics of plasticity and memory proved independent of the number of substrate molecules in our model (not shown; see Equations 6, 10–11, and 15). Conversely, they strongly depend on enzymes characteristics. Decreasing the number of enzyme molecules globally lengthens aKP dynamics: both plasticity time constant and storage lifetime increase. For instance, the timescale of plasticity increases from a fraction of a second to dozens of seconds, whereas memory lifetime increases from days to years (Figure 3B). Conversely, the nonlinearity of enzyme activation has opposite effects on plasticity and memory dynamics. Increasing the Hill number for calcium activation dramatically extends memory lifetime, up to several decades (Figure 3C). Interestingly, even with a Hill coefficient of 1, our model predicts that memory is still a natural property of aKP cycles, with a time constant of ~1 min. By contrast, varying the Hill coefficient in a large range leaves plasticity dynamics virtually unchanged, with a time constant of ~1 s (Figure 3C). Enzyme activation may therefore be of particular importance to mnemonic processes by setting storage lifetime, given a fixed timescale for plasticity induction. Remarkably, these dynamics are obtained using realistic ranges of these enzymatic parameters, and cover the timescales observed in neurons for plasticity induction and short- and long-term memory ([6,8,19,25,28–30,32,39–41,43,53–62]; see Discussion). Moreover, these results indicate that tissue-specific heterogeneity of aKP cycle characteristics may account for the diversity of temporal properties of cellular mnemonic processes among nervous structures.

### Robustness to Molecular Turnover

**Figure 4:**
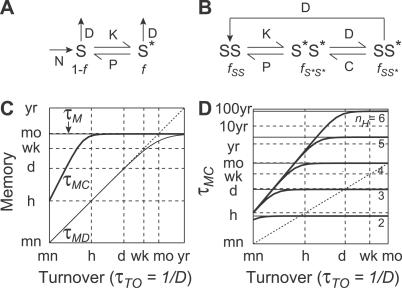
Robustness to Molecular Turnover (A) Reaction scheme of a simple extension of the aKP model incorporating molecular turnover of substrate molecules with degradation rate *D* (see Methods). (B) Reaction scheme of the aKP model incorporating molecular turnover and the protective mechanism proposed by Crick [51] (see Methods). (C) Memory time constant as a function of the time constant of turnover (*τ_TO_* = *1/D*) in the absence of turnover (*τ_M_*, labeled thin line), in the presence of turnover with no protective mechanism (*τ_MD_*, thin curve), and in the presence of turnover and the protective mechanism (*τ_MC_*, thick curve). *n_H_* = 4. (D) Memory time constant in the absence of turnover (thin lines) and in the presence of turnover and the protective mechanism (thick curves) for different values of *n_H_*.

Molecular turnover is susceptible to deteriorating information storage in any molecular implementation of memory states. In principle, this is the case in aKP cycles, since turnover would be assumed to remove both S* and S molecules and incorporate new S molecules, producing a permanent net decrease of the phosphorylated fraction *f*. Turnover should therefore affect information coding (the value of *f*) as well as memory duration (the rate of *f* decrease), and we quantitatively evaluated its impact on aKP cycles. In a straightforward extension of the aKP model, we represent turnover as a combination of first-order degradation of S* and S molecules (with rate constant *D*) and incorporation of new S molecules (with rate *N*, Figure 4A; note that assuming incorporation of S* molecules instead does not change the conclusions below). This scheme is formally equivalent to a supplementary dephosphorylation reaction with rate constant *D* that adds to the phosphatase activity. The memory time constant then reads *τ_MD_* = 1 / (*K* (*Ca_0_*) + *P* (*Ca_0_*) + *D*) (Figure 4A; see Methods, Equation 27). Typically, turnover time constants range from minutes to weeks for proteins [50]. Our model shows that when the turnover time constant (*τ_TO_* =*1* / *D*) is smaller than the memory time constant in the absence of turnover (*τ_M_* = 1 / (*K* (*Ca_0_*) + *P* (*Ca_0_*)); see Methods, Equation 15), memory duration is limited by the time constant of turnover: *τ_MD_* ~ *τ_TO_* (Figure 4C; *τ_MD_* follows the minor diagonal). By opposition, turnover has no impact on memory when *τ_TO_* is larger than *τ_M_* (Figure 4C). Thus, the turnover time constant *τ_TO_* sets the upper limit of memory duration in aKP cycles. Without further assumptions, our model therefore predicts that aKP cycles can at best maintain information up to several weeks—the upper bound for neuronal protein degradation time constant [50]—whatever the Hill number and number of enzyme molecules considered (see Figure 3B and 3C). This value of several weeks encompasses a large range of memory processes, from very short-term to long-term forms of cellular memory [54,63].

Crick [51] has proposed a general protective mechanism against the ravages of turnover. We emphasize that this mechanism is speculative and has not been justified experimentally yet. However, it is effective for multimeric substrates and major molecular targets of KP cycles, such as transmitter-gated synaptic receptors or membrane ion channels, which are multimers. We therefore quantitatively assessed whether the mechanism proposed by Crick indeed protected memory from molecular turnover in aKP cycles. Here, we study the dimeric case, but similar reasoning can be applied to the general multimeric case [51]. In the dimeric case, the aKP cycle interconverts SS and S*S* while turnover is assumed to replace one monomer at a time, transforming S*S* into SS* and SS* into SS. Cricks' proposal further assumes a reaction step that specifically converts SS* to S*S* with rate *C* (Figure 4B). This step might, for instance, be catalyzed by a constitutively active kinase that would specifically recognize SS* and convert it to S*S* (Figure 4B). Alternatively, it might simply represent a spontaneous conformational transition from an energetically unstable SS* state to a more stable S*S* state. This extended version of our model admits a single steady state. The SS* fraction remains extremely small so that its impact on the phosphorylated fraction is negligible (see Methods, Equation 36). Memory is dominated by a single time constant *τ_MC_* (see Methods, Equation 37), and simulations show that the memory time constant is unaffected when the turnover time constant *τ_TO_* is larger than 1 h (Figure 4C; *n_H_* = 4). At lower turnover time constants, *τ_MC_* remains much larger than *τ_TO_*, and increases rapidly with *τ_TO_*; for example, *τ_MC_* is ~1 h with *τ_TO_* = 1 min, ~1 d with *τ_TO_* = 5 min, and more than 2 wk with *τ_TO_* = 30 min (Figure 4C).

We confirmed that the robustness to turnover provided by the protective mechanism was effective over a large range of memory durations by varying the Hill number, *n_H_*, which has a strong impact on the memory time constant (see Figure 3C). As can be seen on Figure 4D, for all *n_H_*, memory degrades with low turnover time constants, but the limit imposed by turnover on the time constant for memory, *τ_MC_*, is rapidly relieved with increasing values of *τ_TO_*: 1 h with *τ_TO_* = 1 min, more than 2 wk with *τ_TO_* = 30 min, and more than 6 mo with *τ_TO_* = 1 h (Figure 4D). For turnover time constant values superior to 1 d, turnover has a virtually null impact on memory (for *n_H_* up to 6; Figure 4D). Therefore, our simulations show that the protective mechanism proposed by Crick [51] can provide an effective mean to generate short- to very long-term memory in the presence of turnover, with time constants compatible with experimental observations.

### Robustness to Basal Calcium Variability

**Figure 5:**
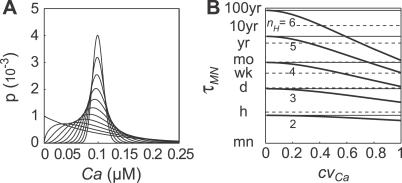
Robustness to Basal Calcium Variability (A) Calcium fluctuations modeled as gamma distributions with mean *Ca*_0_ = 0.1 μM and standard deviations corresponding to coefficient of variation, *cv_Ca_*, in the range 0.01–1. (B) Memory time constant in the absence (thin lines) and presence (thick lines) of calcium fluctuations with gamma distribution, as a function of the coefficient of variation of calcium fluctuations, *cv_Ca_*, for different values of *n_H_*.

The dynamics time constant is a steep function of calcium at low concentrations (see Figure 3A). Consequently, memory duration may be altered by random fluctuations of basal calcium due to spontaneous calcium channel openings or sparks originating from calcium-induced calcium release mechanisms. Little is known about the statistical features of basal calcium fluctuations in vivo. We therefore evaluated memory robustness in the aKP cycle model by computing the average memory time constant using calcium fluctuations that ranged from very peaked to broadly shaped distributions (i.e., with coefficient of variation, *cv_Ca_*, ranging from 0.01 to 1; Figure 5A). We iterated this computation for different values of the Hill number to assess memory robustness to basal calcium fluctuations over a large range of memory timescales. Our results show that memory duration decreases as *cv_Ca_* increases (Figure 5B). This degradation is most prominent for large memory durations (high Hill numbers), but is limited to an order of magnitude or less for mild levels of dispersion of the basal calcium distribution, even at high Hill numbers (Figure 5B; *cv_Ca_* < 0.5). This limited effect is explained by the fact that calcium excursions above *Ca*_0_ that decrease the mean memory time constant (see Figure 3A) are counterbalanced by fluctuations below *Ca*_0_, which increase memory duration. Finally, even very noisy basal calcium distributions cannot abolish memory in aKP cycles: memory duration still ranges from less than an hour to more than a month with *cv_Ca_* = 1 (Figure 5B).

### Plasticity Rules

**Figure 6:**
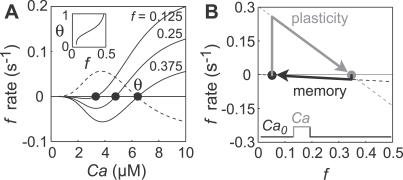
Plasticity and Memory as a Single Dynamic Process (A) *f* rate versus *Ca* in the deterministic model with standard parameters (solid lines) and *K_P_* = 6 μM*, K_K_* = 3 μM (dashed line). Inset: modification threshold *θ* versus *f* with standard parameters. (B) In the aKP cycle model, a saturating *Ca* pulse shifts the single steady state from *f*_∞_(*Ca*_0_) (black circle) to *f*_∞_(*Ca*) (gray circle). *f* converges toward *f*_∞_(*Ca*) at a high rate (gray solid trajectory) along the *f* rate function (gray dashed line). After the pulse, the new *f* value is memorized because *f* relaxes back toward *f*_∞_(*Ca*_0_) at a low rate (black solid trajectory) on the *f* rate function (black dashed line). The rate function at *Ca*_0_ was multiplied by 10^6^ for clarity as it otherwise nearly merges with the *f*-axis (thin line).

If the operation of aKP cycles constitutes a core step in the generation of plastic modifications, as suggested by above results, then aKP cycles should ultimately be able to reproduce the plasticity rules observed experimentally after conditioning protocols. In our model, the phosphorylation rate, *df/dt*, reflects the sign and amplitude of plastic modifications in the neuronal property controlled by *f* (e.g., a synaptic weight or an intrinsic conductance). Experimentally, these modifications are commonly monitored as a percentage change in plasticity experiments (see [16]). We find that plastic modifications in our model display a biphasic pattern as a function of calcium concentration, whatever the *f* value considered (Figure 6A): the *f* rate is negative at low *Ca*, whereas higher *Ca* yields positive *f* rates. In the context of long-term synaptic plasticity, these two domains would, for example, correspond to LTD and LTP, respectively. This pattern accounts for activity-dependent modifications of hippocampal and cortical synaptic weights (Bienenstock, Cooper, and Munro's theoretical “BCM” rule) [16,17,64], or of intrinsic properties [39,40].

In Figure 6A, we have separated the two *Ca* domains of plasticity by a “modification threshold,” *θ*, that characterizes bidirectional plasticity. We find that *θ* increases with *f* (Figure 6A inset). The *f* value, in turn, results from previous activity (i.e., history). Therefore, plasticity in aKP cycles in the model accounts for history-dependent sliding of the modification threshold that has been observed experimentally in synaptic and intrinsic plasticity [16,40,53]. In the model, repetitive high calcium signals become less and less capable of increasing *f* further, because a right-shift in *θ* occurs, which decreases the domain of positive *f* rates. The exactly opposite mechanism applies to repetitive low calcium signals. Hence, *f* is confined within a limited range of values (see Figure 2A and Methods, Equations 13 and 14). Thus, plasticity is saturable in the aKP cycle model, as demonstrated experimentally for synaptic plasticity [56]. Finally, we observe that the two domains of plasticity are inverted when the phosphatase activates at higher calcium concentration than the kinase (Figure 6A, dashed line). Thus, plasticity rules that operate “inversely” to the BCM rule can also be accounted for by aKP cycles. Such rules have also been described experimentally in homeostatic forms of neuronal plasticity or at specific synapses [65,66]. Together, these results show that the operation of aKP cycles embodies a large range of the plasticity rules that are observed in neurons [16,17,39,40,65].

## Discussion

Numerous molecules have been implicated in the induction and maintenance of activity-dependent modifications in neurons, but the mechanisms underlying these processes remain obscure [67]. The idea dominates, though, that maintenance of memory states in neurons reflects the stability of steady states in underlying biophysical or biochemical systems. A common property of autoactivating systems, bistability [2,10,20,21,24], has been pointed out as the possible basis for maintenance of binary plastic modifications [25,26]. Similarly, it has been proposed that graded persistent firing that characterizes short-term memory reflects multistability of neuronal activity [68,69]. However, two essential aspects of information storage in neurons are inconsistent with the idea that stability underpins memory states. First, graded storage, which is found in a large set of neuronal plastic rules [27–39,41], cannot be achieved by bistable mechanisms. Multistable systems can store information in a pseudograded manner, but they rely on distributed computations within complex neural networks or cellular architectures that do not fit the subcellular scale of neuronal plasticity [68,69]. Second, the maintenance of plastic modifications as memory states displays finite lifetimes in neurons (from seconds to months) that are simply not compatible with indefinitely stable steady states ([54,63]; but see [22]). A proper description of neuronal information storage therefore requires alternative—or additional—principles to account for memory states with realistic temporal and coding properties.

Based on the aKP cycle model we have investigated, a principle of dynamic storage emerges that departs from the stability paradigm that has dominated hitherto. Central to this new principle is the idea that plasticity and memory (i.e., the induction of activity-dependent modifications and their maintenance as memory states) are two complementary aspects of a single dynamic storage process whose rate is governed by neuronal activity. In the model, the phosphorylated substrate fraction displays a single stable steady state determined by intracellular calcium concentration, which in turn reflects ongoing neuronal activity. During significant neuronal activity, calcium signals activate enzymes and induce substantial reaction rates. In this case, the system state is plastic: it rapidly converges to the new steady-state value dictated by activity (Figure 6B, gray trajectory). In the opposite case, at basal calcium concentration, reactions are nearly frozen due to the virtual lack of enzyme activation, and the system converges extremely slowly to the rest steady state (Figure 6B, black trajectory). Therefore, the stored state is retained over large timescales, and the system behaves as a “leaky” memory device.

We have conducted a brief meta-analysis of previous results to obtain a summarized view of the time constants for the induction and maintenance of plastic modification in neurons (Table S1). Although this table is not exhaustive, our goal here was to derive an estimate of the physiological magnitude of these values for comparison with those obtained in our model. The kinetics of plasticity and memory depend on the animal species and age, the neuronal type, the preparation (in vivo, slice, or cultured neurons), and the experimental procedure used [70]. We have therefore included these parameters in Table S1.

Experimentally, plastic modifications are induced by stimulations that extend over a large range of durations depending on the type of plasticity studied and the protocol used (Table S1). Often, electrical stimulations of a few seconds are sufficient to induce plasticity [6,19,25,28,30,32,39–41,43,53–56]. In many cases, however, in particular for synaptic plasticity, stimulation protocols extend over much larger periods, from minutes to days, because stimulations must be repeated and/or spaced to be efficient. However, independently of their temporal extent and the type of stimulation they use, plasticity protocols commonly consist of a few hundred elementary stimulating pulses or trains (Table S1). The effective duration of the stimulation activating downstream signaling pathways can be estimated by summing the duration of the postsynaptic potentials triggered by individual stimulations. Postsynaptic depolarizations typically last ~10 ms upon elementary stimulations, so that the effective duration of the stimulation of plasticity protocols ranges from a fraction of a second to dozens of seconds (Table S1; for synaptic plasticity, this timescale is coherent with the fact that direct calcium stimulation for 2 s is sufficient to trigger LTP [43]). Remarkably, this range appears to be consistent across animal species and ages, nervous structures (hippocampus, cortex, cerebellum, amygdala), plasticity types (intrinsic, synaptic), and experimental preparations (in vivo, culture, slices) and conditions. Based on the estimate of the effective time of stimulation of neurons, no particular nervous structure emerges as a “fast” or “slow” learner from the data we have summarized. However, these data are not exhaustive, and differences in experimental protocols may hide fine differences in learning rates. In our model, this range arises as a natural property of aKP cycles and is quantitatively accounted for by the number and nonlinearity of aKPs present in neuronal compartments. Synaptic scaling, a form of plasticity that requires dozens of hours to be recruited [33], stands as a notable exception and may rely on different regulatory processes. Finally, the experimental requirement for spacing stimuli over time is not explained by aKP cycle dynamics (plastic modifications in our model solely depend on the cumulative duration of stimuli). This temporal constraint on inputs might alternatively represent a limitation of our model, or reflect the presence of additional gating mechanisms specifying the neural activity patterns eligible for plasticity, which could be situated upstream or downstream of aKP cycle operation.

Obtaining an overview of the physiological time constants for cellular memory is difficult because monitoring the maintenance of plastic modifications over long periods of time is challenging. Hence, most experiments are conducted for less than 1 h, and only a few studies last long enough to reveal the decay of plastic modifications (see Table S1; data with a *τ* symbol in the maintenance column). Some forms of short-term presynaptic plasticity at Schaffer collateral–CA1 synapses have been demonstrated to have a time constant of 20 s [54] or 20 min [55]. This difference of time course may reflect genuine distinct plastic processes at the same synapse or may arise from differences in experimental procedures (i.e., ages of rats, type of preparation, incubation temperature and duration, recording temperature). With regard to long-term plasticity, several experimental factors can potentially affect the stability and reliability of memory kinetics. Sakijumar et al. [70] have summarized essential criteria that must be respected in vitro to avoid these problems: incubation delays of more than 2–3 h to recover metabolic homeostasis (i.e. second messengers, enzymatic activity, metabolites, and protein phosphorylation levels; [71,72]) and incubation and recording temperatures larger than 32 °C (i.e., for mammals; [73]). None of the in vitro data presented in Table S1 completely fulfill these criteria, and we shall therefore only consider in vivo data. Most of the corresponding studies have been carried out in the hippocampus and display cellular memory time constants that range from hours [29,57] to days [8,57,58] and up to weeks and months [58]. In one case, a form of LTP that was stable for a year has also been described [60]. Finally, a form of LTP with a time constant of weeks has also been found in the cerebral cortex [61]. Similarly, homosynaptic and heterosynaptic forms of LTD present time constants of several days to weeks, respectively [59,62]. Together, these data show that different cellular forms of memory span a very large range of time constants, from seconds to more than a year. According to our model, this range could reflect physiological variability in the biophysical parameters of aKP cycles in different nervous cells or tissues.

Two essential features of memory arise as natural correlates of the principle of dynamic storage in the aKP model. First, information storage is graded, so that several bits of information can be stored. Precisely, storage capacity increases with the number of substrate molecules, from binary and coarse-grained coding at synapses to smoother coding (i.e., dozens of memory states) in large compartments such as somata or large dendritic segments, consistent with experimental observation [52]. Thus, aKP cycles possibly underlie binary LTP observed at CA3–CA1 synapses [25,26] as well as graded plastic modifications of synaptic and intrinsic properties that are not accounted for by prevailing bistable models [27–41]. Note that graded memory may alternatively rely on other molecular mechanisms. For instance, a finely tuned population of bistable elements can theoretically store graded information (see [74] for the principle, although its biophysical implementation is challenging). As a second correlate, the aKP model exhibits finite memory. The present model therefore departs from previous models of plasticity induction that do not account for the maintenance of plastic modifications (i.e., memory [11,12,14,15,48,49]). In such models, plastic modifications vanish very rapidly because the time constant for memory is limited, not exceeding that of plasticity by a factor of ten (e.g., [49]). As a consequence, a constant level of calcium input is required to maintain plastic modifications, violating the first criteria for memory: plastic modifications should last after the offset of the stimulus that induced them [12,49]. Our model also departs from models where an artificial time constant is arbitrarily introduced to obtain desirable memory properties [13]: the time constant for memory in our model is solely determined by experimentally constrained biophysical parameters (see Methods). In particular, we have shown that memory lifetime decreases with increasing numbers of enzyme molecules. Miller et al. [22] recently proposed a CaMKII model in which memory degrades due to stochastic resets of the switch. In this model, memory lifetime increases with the number of enzyme molecules, in striking opposition to our prediction. This dependence constitutes a possible criterion to decide between mechanisms underpinning synaptic plasticity and could be tested experimentally.

A mechanism has been developed recently that could account for graded intrinsic plasticity [52]. However, gradation—and infinite stability—of memory are tautologically ensured in this model by the assumption that plastic modifications are exactly zero (*dX/dt* = 0) in an intermediate range of calcium concentration. Testing whether this hypothesis is biophysically grounded or purely phenomenological remains to be done. In parallel, receptor clustering was recently proposed as an alternative mechanism yielding finite duration memory and graded plasticity [75]. However, it has yet to be determined whether biophysical processes can underlie this mechanism [75]. Moreover, memory lifetime grows exponentially with cluster size (i.e., with synaptic strength): strong synapses last orders of magnitude longer than weak ones, which may induce detrimental biases in memory representations. In addition, this model predicts that the amplitude of plastic modifications increases with synaptic strength, in disagreement with experimental observations [18,56,76].

To ensure that memories are maintained over the long term, information storage in neurons should be robust to different sources of degradation, including the passage of time, turnover, stochastic fluctuations of molecular processes, and memory corruption through the storage of irrelevant ongoing activity. The present model shows that aKP cycles can underlie memory in the presence of turnover, with durations ranging up to the turnover maximal time constants (i.e., weeks [50]). Moreover, we have shown that the speculative mechanism proposed by Crick [51] restores memory timescales that are compatible with animal and human memory even in the presence of molecular turnover. This mechanism has not yet received physiological justification, but its potential importance is considerable. Indeed, it offers an alternative to molecular switches for long-term maintenance of plastic modifications for a large class of multimeric aKP substrates that represent paramount targets of plastic processes (e.g., transmitter-gated synaptic receptors and membrane ion channels). Alternatively, network reactivation during conscious experiences or sleep could activate consolidation mechanisms and stabilize memory states in the long term [77].

Our simulations show that information coding by aKP cycles is reliable even in individual spines, where stochastic fluctuations are maximal. Moreover, in the presence of basal calcium fluctuations, memory degrades to a limited extent for mild variability and can persist up to a month in the presence of very noisy basal calcium distributions. The present model behaves as an integrator (i.e., the storage of new inputs progressively diminishes the influence of previous ones). This can be seen as a drawback of aKP cycles, because the lifetime of a stored input—its effective memory time constant—is decreased by newly stored information. However, all models of memory face this dilemma, and aKP cycles clearly outperform bistable mechanisms in which every transition switch is a reset that totally erases previously stored information. Additional mechanisms may improve protection from memory erasure in aKP cycles. For instance, the modulation of enzyme activation by a second messenger that transduces neuromodulatory or contextual influences could constitute a possible way to gate activity-dependent plastic modifications and to avoid memory corruption by irrelevant activity. Alternatively, several aKP cycles may operate in concert to provide a biophysical implementation of the metaplastic processes that allow memory protection in a recent cascade model of plasticity and memory [78].

Our simulations show that aKP cycles account for critical features of neuronal plasticity. In the present model, the calcium dependence of plasticity is bidirectional, a ubiquitous property of synaptic and intrinsic plasticity [16–18,29,31,39,40]. This property is shared by other KP models [11–15,48,49]. However, the calcium dependence of plasticity in these models corresponds to the steady state of the plastic variable (i.e., *w_∞_*(*Ca*) for a synaptic weight *w*). On the contrary, our model predicts that this calcium dependence reflects the derivative of the plastic variable (i.e., *dw/dt*(*Ca*)). This prediction makes sense because plastic modifications revealed by experimental protocols correspond to variations of the weight from its value before plasticity induction. Mathematically, such variations can be interpreted as the differential of *w*, *dw*, which is proportional to the derivative (*dw* = *dw / dt* (*Ca*) × *dt*, where *dt* represents the duration of plasticity induction). These variations have no a priori reason to be linear with the steady-state *w_∞_*(*Ca*) (our model is one example of this case). To our knowledge, this prediction is new and distinguishes the aKP model from previous models of plasticity induction [12–15,48,49]. Because plasticity is bidirectional in the aKP model, a model coupling an aKP pathway to action potential and N-methyl-D-aspartate receptor descriptions would naturally yield spike-timing dependent plasticity [18,19] in a way similar to previous models [14,15]. Moreover, the present model accounts for activity and history dependence of plasticity in neurons [16,17,40,53]. None of these properties are accounted for by current biophysical bistable models [21,22], whereas they stem from ubiquitous features of KP cycles in the present model (namely the absence of kinase autoactivation, and the dependence of both kinase and phosphatase activation on activity). In addition, plastic modifications display time constants ranging from fraction of a second to dozens of seconds in the aKP model and are consistent with experimental observations, contrary to that in bistable models, which exhibit much longer timescales for plasticity, from dozens of minutes to hours at best [21,22].

To our knowledge, the aKP model is the first to provide a coherent framework that links the induction and maintenance of activity-dependent plastic modifications and accounts for their quintessential features. The model relies on ubiquitous properties of phosphorylation cycles: feed-forward signaling and cooperative activation of enzymes. No specific molecular mechanisms are hypothesized (e.g., autoactivation) and the model behavior proves extremely robust to parameter variations, suggesting that aKP cycles are mechanically responsible for the emergence of neuronal mnemonic properties. Other important enzymatic cycles share similar properties (e.g., *S*-nitrosylation, alcylation; [79,80]), and we believe that the principle we propose may be widespread in mnemonic processes. In our mind, however, the principles of stability and dynamic control are not mutually exclusive. Pathways with binary and graded properties may regulate distinct plastic processes, or alternatively cooperate, to respectively trigger and determine the extent of a common process, for example. Finally, our results constitute an additional example where a simple signaling pathway suffices for local storage of information at the subcellular scale [10–12,21,22,75], supporting the view that local posttranslational protein modifications represent crucial instructive mechanisms underlying neuronal plasticity and memory [4].

## Materials and Methods

### 

#### General principles.

To evaluate the implication of aKP cycles in plasticity and memory, we developed both a stochastic and a deterministic (Figure 1A) model of an aKP cycle operating in a subcellular compartment. In these models, the kinase and the phosphatase determine the phosphorylated fraction *f* of a molecular substrate S by controlling the phosphorylation (S→S*) and dephosphorylation (S*→S) reaction rates. *f* must be regarded as a molecular determinant whose dynamics embody neuronal plastic and mnemonic processes. For instance, *f* may represent the fraction of functional membrane receptors in synaptic plasticity or the fraction of functional ionic channels in intrinsic plasticity. Our aim is to assess the potential role of a generic pathway equipped with simple ubiquitous features that are shared by a large class of kinases and phosphases implied in plasticity and memory.

Both enzymes are activated by molecular signals of neuronal activity. Depending on KP enzymes, the activating signal could be a diffusible second messenger or an intermediate signaling protein or enzyme. Here, the kinase we consider is intended to be generic of a large class of kinases that are directly or indirectly activated by calcium and regulate plastic and mnemonic processes in many neurons. This class includes PKA, PKC, and MAPK. Activation of these kinases by calcium—either directly or indirectly through successive steps within their respective pathways—is cooperative, with a large range of effective Hill coefficients. The Hill coefficient for PKCγ activation is usually 1, but values up to 4 can be found in the literature [81,82]. MAPKs present effective Hill coefficients in the range of 2.5–9 due to nonlinearity integration through the MAPK cascade [83]. In the PKA pathway, calcium induces the production of cAMP, which activates PKA with Hill coefficients ranging from 2 to more than 10 [84,85]. In the present model, we use a conservative effective Hill coefficient of 4 as the standard value. Similarly, we take a Hill coefficient of 4 for the phosphatase, in agreement with values found experimentally for calcineurin [86,87].

In the present work, memory relies on the negligible activation of the kinase and phosphatase at basal calcium concentration. Experimental evidence shows that PKC presents low to virtually null activation at basal calcium concentration in the absence of exogenous DAG, the exact level of activation being dependent, among other factors, on the particular PKC isoenzyme and the substrate molecule considered [81,82,88–90]. In a similar manner, PKA and MAPK [83–85], as well as calcineurin [86,87], display negligible activity at basal activation. As for any memory model that relies on the specific activation of phosphorylation cycles, cross-reactivity could also increase nonspecific phosphorylation and dephosphorylation at basal calcium, thus impinging on memory. However, several biochemical mechanisms might be at work in vivo to control these effects, including compartmentalization, specific inhibition, or allosteric regulation of nonspecific kinases/phosphatases by other effectors. In particular, the confinement of kinases and phosphatases by scaffolding proteins [91,92] may constitute an effective mechanism to prevent cross–reactivity, as proposed for example by Lisman and Zhabotinsky [42] in the case of the CaMKII switch model [21]. Modeling such interactions remains extremely difficult in practice because precise data are lacking to explicitly design such models and quantify the exact impact of compartmentalization on cross-reactivity. Modeling these interactions is therefore largely out of the scope of the present study.

Many substrates are regulated by the aKPs considered in the present model, among which are many ligand-gated membrane receptors (e.g., GABA-A [93] and amino-3-hydroxy-5-methyl-4-isoxazolepropionic acid (AMPA) receptors [94,95]) and voltage-gated ionic channels (e.g., sodium [96] and potassium channels [97]).

Figure 1B displays the effective calcium activation functions of the kinase and phosphatase in the model, using standard parameters. Varying the parameters over wide ranges to evaluate the impact of experimental variability and to account for aKP cycles with different parameters, yielded simulations with qualitatively similar results (i.e., the model behavior was very robust to parameter variations).

#### Stochastic model.

Because plastic and mnemonic processes take place in subcellular compartments where very small numbers of molecules can be encountered, fluctuations of the reaction kinetics are expected to be significant [98]. To evaluate their influence, we simulate the aKP cycle using the exact stochastic simulation algorithm (SSA) from Gillespie that describes the stochastic occurrence of individual phosphorylation and dephosphorylation reaction events [99]. This allows us to analyze the incidence of stochastic fluctuations of the phosphorylated substrate fraction *f* on the capacity of aKP cycles to encode and memorize the information contained in upstream signals. The reaction scheme we study comprises the phosphorylation reaction (S→S*) with propensity

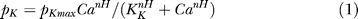
and the dephosphorylation reaction (S*→S) with propensity

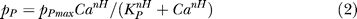
where *Ca* is the intracellular free calcium concentration, *nH* the Hill coefficient, *p_Kmax_* and *p_Pmax_* denote the maximal reaction propensities (at infinite *Ca*), and *K_K_* and *K_P_* denote the half-activation calcium concentrations for the phosphorylation and dephosphorylation reactions. To estimate the *p_max_* of enzymes, let us consider a single enzyme molecule (i.e., a kinase or a phosphatase molecule) and a single substrate molecule (i.e., an S or an S* molecule, respectively) in a subcellular compartment. One has

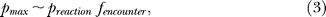
where *p_reaction_* is the reaction probability upon each enzyme/substrate encounter and *f_encounter_* the average frequency of enzyme/substrate encounters. *f_encounter_* can be estimated

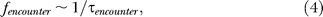
where *τ_encounter_* is the average time for the single enzyme molecule to encounter the single substrate molecule. Considering the compartment and the substrate as 3-D spheres of radius *r_C_* and *r_S_*, respectively, one has [100]

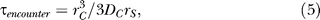
where *D_C_* is the coefficient of diffusion of the enzymes in the subcellular compartment.

Now, for *N_E_* enzymes in the compartment, *τ_encounter_* is divided by *N_E_* so that

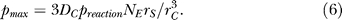



The literature allows us to estimate the parameters values of *D_C_*, *p_reaction_*, *N_E_*, *r_S_*, and *r_C_* to derive a common value of *p_max_* for kinases and phosphatases so that we set *p_Kmax_* = *p_Pmax_* = *p_max_*, assuming that *N_K_* = *N_P_* in the considered compartment. The phosphorylated fraction *f* is computed as *f* = *k* / *N_S_*, where *k* is the number of phosphorylated molecules.

#### Deterministic model.

We derived a deterministic mean-field approximation of the stochastic described model above to obtain a tractable analytical representation of the aKP cycle operation, using mass-action law modeling of the macroscopic reaction scheme (Figure 1A). In this scheme, the dephosphorylated substrate (concentration *S*) is phosphorylated by the kinase with rate *K* while the phosphorylated substrate (concentration S*) is dephosphorylated by the phosphatase with rate *P*. The system thus writes

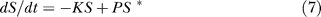


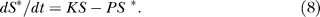



Rewriting this system as a function of *T* = *S* + S*, the total concentration, and *f* = S* / *T*, the phosphorylated fraction, leads to *dT / dt* = 0 (*T* is constant)*,* so that the system eventually consists of a single ordinary differential equation

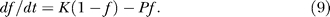

*K* and *P* represent macroscopic reaction rates that are calculated as

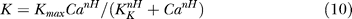
and

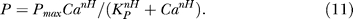

*K_max_* and *P_max_* denote the maximal macroscopic reaction rates that are equal in our case to the propensities *p_Kmax_ and p_Pmax_*.

The model admits a single stable steady state that depends on *Ca*

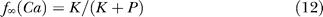
that is bounded between

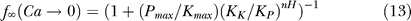
and

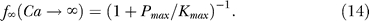

*f* converges exponentially towards *f_∞_* (*Ca*) with a calcium-dependent time constant

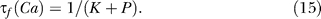



#### Fluctuations and information coding.

We use the mesoscopic approach of Berg and colleagues [101] to analytically describe the fluctuations of *f* due to the finite number of enzyme molecules and substrates. At steady state, the probability *p* (*k*|*Ca*) that *k* substrates are phosphorylated for a given *Ca* value obeys the master equation This distribution is used to compute the mean *f* value and its variance

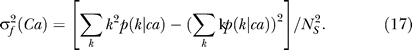
The corresponding coefficient of variation, *cv*, is calculated as the average of σ*_f_* /*f*_∞_ over the *Ca* range 2–10 μM. Note that, provided *N_K_* = *N_P_*,

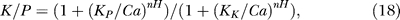
so that *p*(*k*|*Ca*) and *f* random fluctuations do neither depend on *N_K_* nor on *N_P_* in this case. We use information theory to compute the mutual information I[*k*;*Ca*] that represents the amount of information that *f* (or *k*) encodes about *Ca*

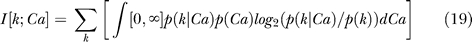
where

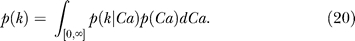



The calcium distribution *p*(*Ca*) is assumed Gaussian with mean (*K_P_* + *K_K_*) / 2 and standard deviation (*K_P_* + *K_K_*) / 3 to mimic average conditions of activity encountered during periods of plasticity.

#### Molecular turnover.

We first modeled substrate turnover as the combination of first-order degradation of S and S* molecules with rate *D* and incorporation of new S molecules as a zero-order process with rate *N* (Figure 4A). The system then writes

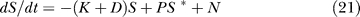


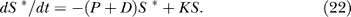
Rewriting this system as a function of *T* = *S* + S*, the total number of substrate molecules, and *f* = S* / *T*, the phosphorylated fraction, leads to

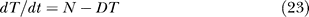


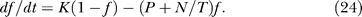



As can be noted, *T* evolves independently of *f*. Once *T* has reached its steady state, *T_∞_* = *N* / *D*, *f* follows

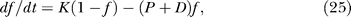
and the system is formally equivalent to modeling turnover as a net dephosphorylation process with rate *D*. The model admits a single stable steady state

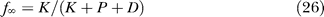
and converges exponentially towards it, with time constant

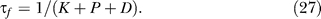



The protective mechanism proposed by Crick [51] was described by the reaction scheme depicted in Figure 4B. In this case, degradation transforms S*S* into SS* dimers and SS* into SS dimers and *T*, the total number of substrate molecules, is constant. The system can thus be written in terms of fractions

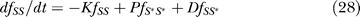


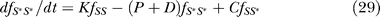


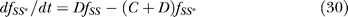
where *C* denotes the rate constant of the protective mechanism. The system can be reduced to

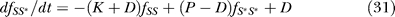


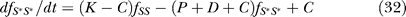


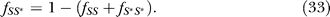



We considered the steady state and time constants of this system at *Ca = Ca*_0_ to assess the impact of turnover. The system was monostable, and the single steady state expresses itself as

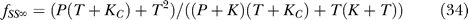


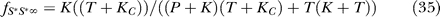


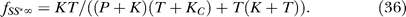



Numerically, *f_SS_* ^*^ *_∞_* remained minute (10^−5^), so that the presence of SS* had a negligible impact on the phosphorylated fraction f_S*S*_.

The system dynamics evolved exponentially with two time constants, but the larger one by far dominated the dynamics and read as

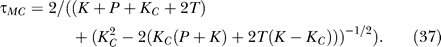



In our simulations, we considered that the protective mechanism converts SS* into S*S* with a rate constant of the order of the phosphatase and kinase of the aKP cycle, as could be expected if this reaction was catalyzed by a constitutive kinase.

#### Basal calcium fluctuations.

The memory time constant of the aKP cycle model was computed in the presence of fluctuations in basal calcium concentration as

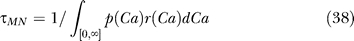
where *r* is the *f* rate (s^−1^) and *p* the probability density function describing calcium fluctuations around the mean basal calcium value *Ca*_0_. *τ_MN_* was calculated for calcium standard deviations of the distribution, giving coefficients of variations in the range 0.01–1. We checked that the computed time constants matched those obtained from fitting *f* decrease during simulations of the aKP cycle model in the presence of basal calcium fluctuations with similar calcium distributions. Using truncated Gaussian distributions (*Ca* > 0) or gamma distributions with mean basal calcium concentration *Ca*_0_ and identical standard deviations provided similar results.

#### Model parameters.

*n_H_* = 4, *K_P_* = 3 μM, and *K_K_* = 6 μM [81–87]; *Ca*_0_ = 0.1 μM. The coefficient of diffusion of CaMKII was recently estimated to be 2 × 10^−14^ m^2^s^−1^ in the spines of neurons by fluorescence recovery after photobleaching [102]. The molecular weight of aKP enzymes lies in the range of ~50–100 kDa (i.e., an order of magnitude below that of CaMKII [~630 kDa]). Assuming proportionality between the molecular weight and the volume of individual enzymes, we estimated *D_C_* = (10^1/3^) 2 × 10^−14^ ≈ 4 × 10^−14^ m^2^s^−1^ in a spine. The coefficient of diffusion in the soma was estimated *D_C_* = 10^−12^ m^2^s^−1^, as for nonneuronal cell bodies [102]. We estimate *p_reaction_* ~ 0.04 from [103], in which the mean time for dephosphorylation of a single potassium channel by a single phosphatase in membrane patches is 24 times that expected in the case of a diffusion-limited reaction (*p_reaction_* = 1). In the simplest case where neurotransmitter receptors or ionic channels represent the direct substrate of aKP cycles, we estimate the substrate radius *r_S_* = 5 nm; *N_S_*, the number of substrate molecules, are estimated to range from less than ten to hundreds in an individual spine and from hundreds to hundreds of thousands in a soma, as are *N_E_*, the number of enzyme (i.e., kinase or phosphatase) molecules [104]. *r_C_*, the radius of the subcellular compartment, is estimated to range from 0.1–0.5 μm for an individual spine and 1–5 μm for a soma; we deliberately underestimate this last range to account for the fact that aPK cycles operate within subcellular compartments of the soma (e.g., submembrane volumes). Using Equation 6 with these ranges of parameters, we find that *p_max_* ranges 0.03–411 s^−1^ for a spine and 0.001–164 s^−1^ for a soma. Considering conservative, representative parameters for a spine (*r_C_* = 0.25 μm; *N_E_* = 10^2^) and a soma (*r_C_* = 2.5 μm; *N_E_* = 10^4^), we obtain *p_max_* = 0.31 s^−1^ for a spine and *p_max_* = 0.77 s^−1^ for a soma. The time constants for plasticity and memory are arbitrarily defined as the values of *τ_f_*(*Ca*) computed at *Ca* = 10 μM (virtually maximal enzyme activity) and at basal calcium concentration *Ca*_0_ = 0.1 μM, respectively. With the parameters we used, the time constant of plasticity is ~1.63 s for a spine and ~0.65 s for a somatic compartment, consistent with experimental observation that plastic modifications are triggered at the second timescale [6,25,30,39,43]. Unless mentioned, the results illustrated are obtained for a spine, with *N_S_* = 10^2^. The constitutive kinase rate is taken on the same order as that of the other enzymes at maximal activation, *C =* 1 s^−1^ [103]. The degradation rate *D* is set in the range minutes to weeks, as found experimentally [50].

## Supporting Information

Table S1 Experimental Parameters and Properties for the Induction and Maintenance of Plastic Modifications Inc. Delay: incubation delay. Inc. T, Rec T: incubation and recording temperatures in Celsius degrees. Ext. Pre., Freq. Pre., N Pre., Eff. Dur. Pre.: temporal extension, frequency (Hz), number, and effective duration of presynaptic stimuli. Post.: postsynaptic stimulation characteristics. The effective duration of presynaptic stimulation was estimated as the product of the number of presynaptic stimulations by the duration of excitation per unitary stimulus. This duration was estimated to be 0.01 s, the typical time constant of excitatory postsynaptic potentials (EPSPs). This calculation is valid for stimulation frequencies up to the order of 100 Hz. For higher frequencies, successive EPSPs merge within a train, and the duration of the excitation provoked by an individual train was estimated as the sum of the duration of the train (0.02 s for [58–60]; 0.035 s for [8,61]) plus the time constant of an EPSP. Maintenance: duration of the monitored maintenance or time constant (τ symbol) of activity-dependent modifications.
(50 KB XLS)

## Abbreviations

aKP: activity-dependent kinase and phosphatase
CaMKII: Ca2^+^/calmodulin-dependent protein kinase II
EPSP: excitatory postsynaptic potential
KP: kinase and phosphatase
LTD: long-term depression
LTP: long-term potentiation
MAPK: mitogen-activated protein kinase
PKA: protein kinase A
PKC: protein kinase C

## References

[pcbi-0030124-b001] Martin SJ, Grimwood PD, Morris RG (2000). Synaptic plasticity and memory: An evaluation of the hypothesis. Annu Rev Neurosci.

[pcbi-0030124-b002] Bhalla US, Iyengar R (1999). Emergent properties of networks of biological signaling pathways. Science.

[pcbi-0030124-b003] Kandel ER (2001). The molecular biology of memory storage: A dialogue between genes and synapses. Science.

[pcbi-0030124-b004] Routtenberg A, Rekart JL (2005). Post-translational protein modification as the substrate for long-lasting memory. Trends Neurosci.

[pcbi-0030124-b005] Soderling TR, Derkach VA (2000). Postsynaptic protein phosphorylation and LTP. Trends Neurosci.

[pcbi-0030124-b006] Ganguly K, Kiss L, Poo M (2000). Enhancement of presynaptic neuronal excitability by correlated presynaptic and postsynaptic spiking. Nat Neurosci.

[pcbi-0030124-b007] Winder DG, Sweatt JD (2001). Roles of serine/threonine phosphatases in hippocampal synaptic plasticity. Nat Rev Neurosci.

[pcbi-0030124-b008] Malleret G, Haditsch U, Genoux D, Jones MW, Bliss TV (2001). Inducible and reversible enhancement of learning, memory, and long-term potentiation by genetic inhibition of calcineurin. Cell.

[pcbi-0030124-b009] Huang YY, Pittenger C, Kandel ER (2004). A form of long-lasting, learning-related synaptic plasticity in the hippocampus induced by heterosynaptic low-frequency pairing. Proc Natl Acad Sci U S A.

[pcbi-0030124-b010] Lisman JE (1985). A mechanism for memory storage insensitive to molecular turnover: A bistable autophosphorylating kinase. Proc Natl Acad Sci U S A.

[pcbi-0030124-b011] D'Alcantara P, Schiffmann SN, Swillens S (2003). Bidirectional synaptic plasticity as a consequence of interdependent Ca2^+^-controlled phosphorylation and dephosphorylation pathways. Eur J Neurosci.

[pcbi-0030124-b012] Castellani GC, Quinlan EM, Cooper LN, Shouval HZ (2001). A biophysical model of bidirectional synaptic plasticity: Dependence on AMPA and NMDA receptors. Proc Natl Acad Sci U S A.

[pcbi-0030124-b013] Smolen PD, Baxter DA, Byrne JH (2006). A model of the roles of essential kinases in the induction and expression of late long-term potentiation. Biophys J.

[pcbi-0030124-b014] Abarbanel HD, Gibb L, Huerta R, Rabinovich MI (2003). Biophysical model of synaptic plasticity dynamics. Biol Cybern.

[pcbi-0030124-b015] Shouval HZ, Bear MF, Cooper LN (2002). A unified model of NMDA receptor-dependent bidirectional synaptic plasticity. Proc Natl Acad Sci U S A.

[pcbi-0030124-b016] Bear MF (1996). A synaptic basis for memory storage in the cerebral cortex. Proc Natl Acad Sci U S A.

[pcbi-0030124-b017] Ismailov I, Kalikulov D, Inoue T, Friedlander MJ (2004). The kinetic profile of intracellular calcium predicts long-term potentiation and long-term depression. J Neurosci.

[pcbi-0030124-b018] Sjostrom PJ, Turrigiano GG, Nelson SB (2001). Rate, timing, and cooperativity jointly determine cortical synaptic plasticity. Neuron.

[pcbi-0030124-b019] Nishiyama M, Hong K, Mikoshiba K, Poo MM, Kato K (2000). Calcium stores regulate the polarity and input specificity of synaptic modification. Nature.

[pcbi-0030124-b020] Angeli D, Ferrell JE, Sontag ED (2004). Detection of multistability, bifurcations, and hysteresis in a large class of biological positive-feedback systems. Proc Natl Acad Sci U S A.

[pcbi-0030124-b021] Zhabotinsky AM (2000). Bistability in the Ca(2+)/calmodulin-dependent protein kinase-phosphatase system. Biophys J.

[pcbi-0030124-b022] Miller P, Zhabotinsky AM, Lisman JE, Wang XJ (2005). The stability of a stochastic CaMKII switch: Dependence on the number of enzyme molecules and protein turnover. PLoS Biol.

[pcbi-0030124-b023] Hayer A, Bhalla US (2005). Molecular switches at the synapse emerge from receptor and kinase traffic. PLoS Comput Biol.

[pcbi-0030124-b024] Song H, Smolen P, Av-Ron E, Baxter DA, Byrne JH (2006). Bifurcation and singularity analysis of a molecular network for the induction of long-term memory. Biophys J.

[pcbi-0030124-b025] Petersen CC, Malenka RC, Nicoll RA, Hopfield JJ (1998). All-or-none potentiation at CA3-CA1 synapses. Proc Natl Acad Sci U S A.

[pcbi-0030124-b026] O'Connor DH, Wittenberg GM, Wang SS (2005). Graded bidirectional synaptic plasticity is composed of switch-like unitary events. Proc Natl Acad Sci U S A.

[pcbi-0030124-b027] Montgomery JM, Madison DV (2002). State-dependent heterogeneity in synaptic depression between pyramidal cell pairs. Neuron.

[pcbi-0030124-b028] Shi SH, Hayashi Y, Petralia RS, Zaman SH, Wenthold RJ (1999). Rapid spine delivery and redistribution of AMPA receptors after synaptic NMDA receptor activation. Science.

[pcbi-0030124-b029] Heynen AJ, Quinlan EM, Bae DC, Bear MF (2000). Bidirectional, activity-dependent regulation of glutamate receptors in the adult hippocampus in vivo. Neuron.

[pcbi-0030124-b030] Luthi A, Wikstrom MA, Palmer MJ, Matthews P, Benke TA (2004). Bi-directional modulation of AMPA receptor unitary conductance by synaptic activity. BMC Neurosci.

[pcbi-0030124-b031] Tomita S, Stein V, Stocker TJ, Nicoll RA, Bredt DS (2005). Bidirectional synaptic plasticity regulated by phosphorylation of stargazin-like TARPs. Neuron.

[pcbi-0030124-b032] Esteban JA, Shi SH, Wilson C, Nuriya M, Huganir RL (2003). PKA phosphorylation of AMPA receptor subunits controls synaptic trafficking underlying plasticity. Nat Neurosci.

[pcbi-0030124-b033] Turrigiano GG, Leslie KR, Desai NS, Rutherford LC, Nelson SB (1998). Activity-dependent scaling of quantal amplitude in neocortical neurons. Nature.

[pcbi-0030124-b034] Watt AJ, van Rossum MC, MacLeod KM, Nelson SB, Turrigiano GG (2000). Activity coregulates quantal AMPA and NMDA currents at neocortical synapses. Neuron.

[pcbi-0030124-b035] Magee JC, Cook EP (2000). Somatic EPSP amplitude is independent of synapse location in hippocampal pyramidal neurons. Nat Neurosci.

[pcbi-0030124-b036] Smith MA, Ellis-Davies GC, Magee JC (2003). Mechanism of the distance-dependent scaling of Schaffer collateral synapses in rat CA1 pyramidal neurons. J Physiol.

[pcbi-0030124-b037] Royer S, Pare D (2003). Conservation of total synaptic weight through balanced synaptic depression and potentiation. Nature.

[pcbi-0030124-b038] Turrigiano G, Abbott LF, Marder E (1994). Activity-dependent changes in the intrinsic properties of cultured neurons. Science.

[pcbi-0030124-b039] Egorov AV, Hamam BN, Fransen E, Hasselmo ME, Alonso AA (2002). Graded persistent activity in entorhinal cortex neurons. Nature.

[pcbi-0030124-b040] Daoudal G, Hanada Y, Debanne D (2002). Bidirectional plasticity of excitatory postsynaptic potential (EPSP)-spike coupling in CA1 hippocampal pyramidal neurons. Proc Natl Acad Sci U S A.

[pcbi-0030124-b041] Oestreich J, Dembrow NC, George AA, Zakon HH (2006). A “sample-and-hold” pulse-counting integrator as a mechanism for graded memory underlying sensorimotor adaptation. Neuron.

[pcbi-0030124-b042] Lisman JE, Zhabotinsky AM (2001). A model of synaptic memory: A CaMKII/PP1 switch that potentiates transmission by organizing an AMPA receptor anchoring assembly. Neuron.

[pcbi-0030124-b043] Malenka RC, Lancaster B, Zucker RS (1992). Temporal limits on the rise in postsynaptic calcium required for the induction of long-term potentiation. Neuron.

[pcbi-0030124-b044] Chen HX, Otmakhov N, Strack S, Colbran RJ, Lisman JE (2001). Is persistent activity of calcium/calmodulin-dependent kinase required for the maintenance of LTP?. J Neurophysiol.

[pcbi-0030124-b045] Lengyel I, Voss K, Cammarota M, Bradshaw K, Brent V (2004). Autonomous activity of CaMKII is only transiently increased following the induction of long-term potentiation in the rat hippocampus. Eur J Neurosci.

[pcbi-0030124-b046] Cooke SF, Wu J, Plattner F, Errington M, Rowan M (2006). Autophosphorylation of alphaCaMKII is not a general requirement for NMDA receptor-dependent LTP in the adult mouse. J Physiol.

[pcbi-0030124-b047] Ito M (2001). Cerebellar long-term depression: Characterization, signal transduction, and functional roles. Physiol Rev.

[pcbi-0030124-b048] Castellani GC, Quinlan EM, Bersani F, Cooper LN, Shouval HZ (2005). A model of bidirectional synaptic plasticity: From signaling network to channel conductance. Learn Mem.

[pcbi-0030124-b049] Shouval HZ, Castellani GC, Blais BS, Yeung LC, Cooper LN (2002). Converging evidence for a simplified biophysical model of synaptic plasticity. Biol Cybern.

[pcbi-0030124-b050] Droz B, Koenig HL, Biamberardino LD, Di Giamberardino L (1973). Axonal migration of protein and glycoprotein to nerve endings. I. Radioautographic analysis of the renewal of protein in nerve endings of chicken ciliary ganglion after intracerebral injection of (3H)lysine. Brain Res.

[pcbi-0030124-b051] Crick F (1984). Memory and molecular turnover. Nature.

[pcbi-0030124-b052] Fransen E, Tahvildari B, Egorov AV, Hasselmo ME, Alonso AA (2006). Mechanism of graded persistent cellular activity of entorhinal cortex layer v neurons. Neuron.

[pcbi-0030124-b053] Kirkwood A, Rioult MC, Bear MF (1996). Experience-dependent modification of synaptic plasticity in visual cortex. Nature.

[pcbi-0030124-b054] Brager DH, Cai X, Thompson SM (2003). Activity-dependent activation of presynaptic protein kinase C mediates post-tetanic potentiation. Nat Neurosci.

[pcbi-0030124-b055] Volianskis A, Jensen MS (2003). Transient and sustained types of long-term potentiation in the CA1 area of the rat hippocampus. J Physiol.

[pcbi-0030124-b056] Rioult-Pedotti MS, Friedman D, Donoghue JP (2000). Learning-induced LTP in neocortex. Science.

[pcbi-0030124-b057] Racine RJ, Milgram NW, Hafner S (1983). Long-term potentiation phenomena in the rat limbic forebrain. Brain Res.

[pcbi-0030124-b058] Abraham WC, Mason SE, Demmer J, Williams JM, Richardson CL (1993). Correlations between immediate early gene induction and the persistence of long-term potentiation. Neuroscience.

[pcbi-0030124-b059] Abraham WC, Christie BR, Logan B, Lawlor P, Dragunow M (1994). Immediate early gene expression associated with the persistence of heterosynaptic long-term depression in the hippocampus. Proc Natl Acad Sci U S A.

[pcbi-0030124-b060] Abraham WC, Logan B, Greenwood JM, Dragunow M (2002). Induction and experience-dependent consolidation of stable long-term potentiation lasting months in the hippocampus. J Neurosci.

[pcbi-0030124-b061] Trepel C, Racine RJ (1998). Long-term potentiation in the neocortex of the adult, freely moving rat. Cereb Cortex.

[pcbi-0030124-b062] Doyere V, Errington ML, Laroche S, Bliss TV (1996). Low-frequency trains of paired stimuli induce long-term depression in area CA1 but not in dentate gyrus of the intact rat. Hippocampus.

[pcbi-0030124-b063] Abraham WC (2003). How long will long-term potentiation last?. Philos Trans R Soc Lond B Biol Sci.

[pcbi-0030124-b064] Bienenstock EL, Cooper LN, Munro PW (1982). Theory for the development of neuron selectivity: Orientation specificity and binocular interaction in visual cortex. J Neurosci.

[pcbi-0030124-b065] Turrigiano GG (1999). Homeostatic plasticity in neuronal networks: The more things change, the more they stay the same. Trends Neurosci.

[pcbi-0030124-b066] Coesmans M, Weber JT, De Zeeuw CI, Hansel C (2004). Bidirectional parallel fiber plasticity in the cerebellum under climbing fiber control. Neuron.

[pcbi-0030124-b067] Sanes JR, Lichtman JW (1999). Can molecules explain long-term potentiation?. Nat Neurosci.

[pcbi-0030124-b068] Brody CD, Romo R, Kepecs A (2003). Basic mechanisms for graded persistent activity: Discrete attractors, continuous attractors, and dynamic representations. Curr Opin Neurobiol.

[pcbi-0030124-b069] Loewenstein Y, Sompolinsky H (2003). Temporal integration by calcium dynamics in a model neuron. Nat Neurosci.

[pcbi-0030124-b070] Sajikumar S, Navakkode S, Frey JU (2005). Protein synthesis-dependent long-term functional plasticity: Methods and techniques. Curr Opin Neurobiol.

[pcbi-0030124-b071] Whittingham TS, Lust WD, Christakis DA, Passonneau JV (1984). Metabolic stability of hippocampal slice preparations during prolonged incubation. J Neurochem.

[pcbi-0030124-b072] Ho OH, Delgado JY, O'Dell TJ (2004). Phosphorylation of proteins involved in activity-dependent forms of synaptic plasticity is altered in hippocampal slices maintained in vitro. J Neurochem.

[pcbi-0030124-b073] Micheva KD, Smith SJ (2005). Strong effects of subphysiological temperature on the function and plasticity of mammalian presynaptic terminals. J Neurosci.

[pcbi-0030124-b074] Lisman J (1989). A mechanism for the Hebb and the anti-Hebb processes underlying learning and memory. Proc Natl Acad Sci U S A.

[pcbi-0030124-b075] Shouval HZ (2005). Clusters of interacting receptors can stabilize synaptic efficacies. Proc Natl Acad Sci U S A.

[pcbi-0030124-b076] Bi GQ, Poo MM (1998). Synaptic modifications in cultured hippocampal neurons: Dependence on spike timing, synaptic strength, and postsynaptic cell type. J Neurosci.

[pcbi-0030124-b077] Frankland PW, Bontempi B (2005). The organization of recent and remote memories. Nat Rev Neurosci.

[pcbi-0030124-b078] Fusi S, Drew PJ, Abbott LF (2005). Cascade models of synaptically stored memories. Neuron.

[pcbi-0030124-b079] Roth SY, Denu JM, Allis CD (2001). Histone acetyltransferases. Annu Rev Biochem.

[pcbi-0030124-b080] Mannick JB, Schonhoff CM (2002). Nitrosylation: The next phosphorylation?. Arch Biochem Biophys.

[pcbi-0030124-b081] Nishizuka Y (1988). The molecular heterogeneity of protein kinase C and its implications for cellular regulation. Nature.

[pcbi-0030124-b082] Kohout SC, Corbalan-Garcia S, Torrecillas A, Gomez-Fernandez JC, Falke JJ (2002). C2 domains of protein kinase C isoforms alpha, beta, and gamma: Activation parameters and calcium stoichiometries of the membrane-bound state. Biochemistry.

[pcbi-0030124-b083] Huang CY, Ferrell JE (1996). Ultrasensitivity in the mitogen-activated protein kinase cascade. Proc Natl Acad Sci U S A.

[pcbi-0030124-b084] Huang LJ, Taylor SS (1998). Dissecting cAMP binding domain A in the RIalpha subunit of cAMP-dependent protein kinase. Distinct subsites for recognition of cAMP and the catalytic subunit. J Biol Chem.

[pcbi-0030124-b085] Gerlach AC, Gangopadhyay NN, Devor DC (2000). Kinase-dependent regulation of the intermediate conductance, calcium-dependent potassium channel, hIK1. J Biol Chem.

[pcbi-0030124-b086] Stewart AA, Ingebritsen TS, Cohen P (1983). The protein phosphatases involved in cellular regulation. 5. Purification and properties of a Ca2^+^/calmodulin-dependent protein phosphatase (2B) from rabbit skeletal muscle. Eur J Biochem.

[pcbi-0030124-b087] Stemmer PM, Klee CB (1994). Dual calcium ion regulation of calcineurin by calmodulin and calcineurin B. Biochemistry.

[pcbi-0030124-b088] Schaechter JD, Benowitz LI (1993). Activation of protein kinase C by arachidonic acid selectively enhances the phosphorylation of GAP-43 in nerve terminal membranes. J Neurosci.

[pcbi-0030124-b089] Takai Y, Kishimoto A, Iwasa Y, Kawahara Y, Mori T (1979). Calcium-dependent activation of a multifunctional protein kinase by membrane phospholipids. J Biol Chem.

[pcbi-0030124-b090] Long GJ, Rosen JF, Schanne FA (1994). Lead activation of protein kinase C from rat brain. Determination of free calcium, lead, and zinc by 19F NMR. J Biol Chem.

[pcbi-0030124-b091] Smith KE, Gibson ES, Dell'Acqua ML (2006). cAMP-dependent protein kinase postsynaptic localization regulated by NMDA receptor activation through translocation of an A-kinase anchoring protein scaffold protein. J Neurosci.

[pcbi-0030124-b092] Yoshimura Y, Sogawa Y, Yamauchi T (1999). Protein phosphatase 1 is involved in the dissociation of Ca2^+^/calmodulin-dependent protein kinase II from postsynaptic densities. FEBS Lett.

[pcbi-0030124-b093] Poisbeau P, Cheney MC, Browning MD, Mody I (1999). Modulation of synaptic GABAA receptor function by PKA and PKC in adult hippocampal neurons. J Neurosci.

[pcbi-0030124-b094] Snyder GL, Galdi S, Fienberg AA, Allen P, Nairn AC (2003). Regulation of AMPA receptor dephosphorylation by glutamate receptor agonists. Neuropharmacology.

[pcbi-0030124-b095] Roche KW, O'Brien RJ, Mammen AL, Bernhardt J, Huganir RL (1996). Characterization of multiple phosphorylation sites on the AMPA receptor GluR1 subunit. Neuron.

[pcbi-0030124-b096] Hourez R, Azdad K, Vanwalleghem G, Roussel C, Gall D (2005). Activation of protein kinase C and inositol 1,4,5-triphosphate receptors antagonistically modulate voltage-gated sodium channels in striatal neurons. Brain Res.

[pcbi-0030124-b097] Misonou H, Menegola M, Mohapatra DP, Guy LK, Park KS (2006). Bidirectional activity-dependent regulation of neuronal ion channel phosphorylation. J Neurosci.

[pcbi-0030124-b098] Bhalla US (2004). Signaling in small subcellular volumes. II. Stochastic and diffusion effects on synaptic network properties. Biophys J.

[pcbi-0030124-b099] Gillespie DT (1977). Exact stochastic simulation of coupled chemical reactions. J Chem Phys.

[pcbi-0030124-b100] Adam G, Delbrück M, Rich A, Davidson N (1968). Reduction of dimensionality in biological diffusion processes. Structural chemistry and molecular biology.

[pcbi-0030124-b101] Berg OG, Paulsson J, Ehrenberg M (2000). Fluctuations and quality of control in biological cells: Zero-order ultrasensitivity reinvestigated. Biophys J.

[pcbi-0030124-b102] Smith BA, Roy H, De Koninck P, Grutter P, De Koninck Y (2007). Dendritic spine viscoelasticity and soft-glassy nature: Balancing dynamic remodeling with structural stability. Biophys J.

[pcbi-0030124-b103] Bielefeldt K, Jackson MB (1994). Intramolecular and intermolecular enzymatic modulation of ion channels in excised membrane patches. Biophys J.

[pcbi-0030124-b104] Hille B (1992). Ionic channels of excitable membranes.

